# Transmission of SARS-CoV 2 During Long-Haul Flight

**DOI:** 10.3201/eid2611.203299

**Published:** 2020-11

**Authors:** Nguyen Cong Khanh, Pham Quang Thai, Ha-Linh Quach, Ngoc-Anh Hoang Thi, Phung Cong Dinh, Tran Nhu Duong, Le Thi Quynh Mai, Ngu Duy Nghia, Tran Anh Tu, La Ngoc Quang, Tran Dai Quang, Trong-Tai Nguyen, Florian Vogt, Dang Duc Anh

**Affiliations:** National Institute of Hygiene and Epidemiology, Hanoi, Vietnam (N.C. Khanh, P.Q. Thai, H.-L. Quach, N.-A.H. Thi, T.N. Duong, L.T.Q. Mai, N.D. Nghia, T.A. Tu, D.D. Anh);; Hanoi Medical University, Hanoi (P.Q. Thai, T.-T. Nguyen);; Australian National University, Canberra, Australian Capital Territory, Australia (H.-L. Quach, N.-A. H. Thi, F. Vogt);; Ministry of Science and Technology, Hanoi (P.C. Dinh);; Ha Noi University of Public Health, Hanoi (L.N. Quang);; Ministry of Health, Hanoi (T.D. Quang)

**Keywords:** 2019 novel coronavirus disease, coronavirus disease, COVID-19, severe acute respiratory syndrome coronavirus 2, SARS-CoV-2, viruses, respiratory infections, zoonoses, aircraft, transmission, air travel

## Abstract

To assess the role of in-flight transmission of severe acute respiratory syndrome coronavirus 2 (SARS-CoV-2), we investigated a cluster of cases among passengers on a 10-hour commercial flight. Affected persons were passengers, crew, and their close contacts. We traced 217 passengers and crew to their final destinations and interviewed, tested, and quarantined them. Among the 16 persons in whom SARS-CoV-2 infection was detected, 12 (75%) were passengers seated in business class along with the only symptomatic person (attack rate 62%). Seating proximity was strongly associated with increased infection risk (risk ratio 7.3, 95% CI 1.2–46.2). We found no strong evidence supporting alternative transmission scenarios. In-flight transmission that probably originated from 1 symptomatic passenger caused a large cluster of cases during a long flight. Guidelines for preventing SARS-CoV-2 infection among air passengers should consider individual passengers’ risk for infection, the number of passengers traveling, and flight duration.

During the first 6 months of 2020, severe acute respiratory syndrome coronavirus 2 (SARS-CoV-2) spread to almost all countries and infected »4 million persons worldwide (*1*). Air travel is contributing to the extent and speed of the pandemic spread through the movement of infected persons (*2*–*4*); consequently, in March, many countries either completely halted or substantially reduced air travel.

Spread of SARS-CoV-2 across international borders by infected travelers has been well documented (*5*,*6*); however, evidence and in-depth assessment of the risk for transmission from infected passengers to other passengers or crew members during the course of a flight (in-flight transmission) are limited. Although the international flight industry has judged the risk for in-flight transmission to be very low (*7*), long flights in particular have become a matter of increasing concern as many countries have started lifting flight restrictions despite ongoing SARS-CoV-2 transmission (*8*).

The first case of coronavirus disease (COVID-19) in Vietnam was recorded on January 23, 2020; the patient was a visitor from Wuhan, China (*9*). On January 24, Vietnam suspended air travel from mainland China, Hong Kong, and Taiwan and, as the epidemic spread worldwide, gradually expanded travel bans, mandatory quarantine, and testing measures to incoming passengers from other countries (*10*).

In early March, when much of the global community was just beginning to recognize the severity of the pandemic, we detected a cluster of COVID-19 cases among passengers arriving on the same flight from London, UK, to Hanoi, Vietnam, on March 2 (Vietnam Airlines flight 54 [VN54]). At that time, importation of COVID-19 had been documented in association with 3 flights to Vietnam, including a cluster of 6 persons who had index cases and were evacuated from Wuhan; 6 secondary cases resulted from virus transmission in Vietnam (*11*). No in-depth investigations among passengers on those flights were conducted, and no evidence indicated that transmission had occurred during the flights themselves.

Initial investigations of flight VN54 led us to hypothesize potential in-flight transmission originating from 1 symptomatic passenger in business class (the probable index case). We subsequently launched an extensive epidemiologic investigation that involved testing and isolation/quarantine of all traceable passengers and crew members of the identified flight. Our objectives were to estimate the probability that transmission of SARS-CoV-2 occurred on the flight in question and to identify risk factors associated with transmission.

## Methods

We defined cases of SARS-CoV-2 infection according to Vietnam Ministry of Health guidelines in place at the time of our investigation (*12*). Specifically, we defined suspected flight-associated COVID-19 cases as passengers or crew members on board flight VN54 landing in Hanoi on March 2 who reported fever and cough, with or without shortness of breath, during March 1–16. We defined confirmed flight-associated COVID-19 cases as passengers or crew members on flight VN54, regardless whether signs or symptoms developed, who had positive SARS-CoV-2 real-time reverse transcription PCR results from nasopharyngeal swab samples (*13*). Flight-associated cases were considered to have very likely acquired infection on board VN54 and were hence classified as probable secondary cases in this analysis if the following 3 criteria were met: 1) they experienced signs/symptoms 2–14 days after arrival or if they were SARS-CoV-2 positive by PCR 2–14 days after arrival in the absence of signs/symptoms; 2) in-depth investigation did not reveal any potential exposure to SARS-CoV-2 before or after the flight during their incubation period; and 3) they had shared cabin space with the probable index case during the flight (*14*–*17*).

At the time of flight VN54 arrival, all passengers from COVID-19–infected areas, including the United Kingdom, had their body temperature screened by thermal imaging and were required to declare any COVID-19 symptoms; only passengers arriving from China, South Korea, Iran, or Italy were required to undergo SARS-CoV-2 testing and 14-day quarantine. At that time, the use of face masks was not mandatory on airplanes or at airports (*18*).

As soon as the travel history of the probable index case became evident, the passenger list and flight manifest for flight VN54 was obtained from the Bureau of Immigration and the Civil Aviation Administration and sent to all provincial Centers for Disease Control with instructions for local health staff to trace all passengers and crew members of flight VN54. All successfully traced passengers and crew members were interviewed by use of a standard questionnaire, tested for SARS-CoV-2, and quarantined in designated facilities or at home. Any symptomatic person was isolated immediately until the test result was received. In-depth interviews were conducted with all persons with suspected or confirmed flight-associated cases; the specific focus was detecting any potential SARS-CoV-2 transmission events before and after the flight to investigate potential alternative scenarios for transmission other than during the flight. Furthermore, all persons with suspected or confirmed flight-associated cases were asked to identify persons with whom they had had close contact (<2 meter distance for >15 minutes) between arriving in Vietnam and the start of quarantine/isolation. These close contacts were also contacted, tested, and quarantined for 14 days. All persons in quarantine were checked twice daily for clinical signs/symptoms and fever; oropharyngeal swabs were collected on the day of admission, after 3–5 days, and on day 13, unless signs/symptoms developed, in which instance a specimen was collected immediately and the person was isolated and monitored until receipt of the test result.

Initial investigations of the probable index case generated our working hypothesis of in-flight transmission and guided further investigations. In particular, we investigated all possible exposures of all persons with flight-associated cases during their incubation period in relation to the timing of the flight, including locations where flight-associated cases may have crossed paths before and after the flight. To identify factors associated with in-flight infection risks, we calculated risk ratios and 95% CIs.

## Results

### Setting

**Figure 1:**
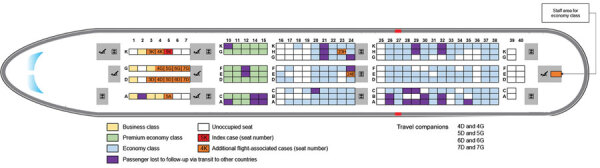
Seating location of passengers on Vietnam Airlines flight 54 from London, UK, to Hanoi, Vietnam, on March 2, 2020, for whom severe acute respiratory syndrome coronavirus 2 infection was later confirmed.

Flight VN54 departed London at 11:10 am local time on March 1, 2020, and arrived in Hanoi at 5:20 am local time on March 2; the nonstop flight lasted about 10 hours. A total of 16 crew members and 201 passengers were on board. The 274 seats on the airplane were divided into business class (28 seats), premium economy class (35 seats), and economy class (211 seats); there were 4 toilets for business and premium economy classes and 5 for economy. The business class was exclusively reserved and separated from the premium economy and economy classes by a service/toilet area (Figure 1). Of the 201 passengers, 21 occupied business (75% seats occupied), 35 premium economy (100%), and 145 economy (67%) seats (Figure 1). Two meals were served, and flight attendants worked in 2 teams, 1 for the business and premium economy sections and 1 for the economy section.

### Investigation of Probable Index Case

A 27-year-old businesswoman from Vietnam, whom we identified as the probable index case (hereafter case 1), had been based in London since early February. Our case investigations supplemented by information obtained from media reports indicated that she had traveled to Italy on February 18 with her sister, who was later confirmed to be SARS-CoV-2–positive in London, and back to London on February 20 to stay with her sister for another 2 nights. On February 22, case 1 and her sister returned to Milan, Italy, and subsequently traveled to Paris, France, for the yearly Fashion Week before returning back to London on February 25. They continued to reside in London until February 29, when case 1 started to experience a sore throat and cough while attending meetings and visiting entertainment hubs with friends. On March 1, she boarded flight VN54. She was seated in business class and continued to experience the sore throat and cough throughout the flight. Her signs and symptoms (fever, sore throat, fatigue, and shortness of breath) progressed further after arrival, and she self-isolated at her private residence in Hanoi and had contact with household personnel only. On March 5, she sought care at a local hospital in Hanoi, where an oropharyngeal swab sample was taken and tested; SARS-CoV-2 infection was confirmed by real-time reverse transcription PCR on March 6. On March 7, three of her household personnel received positive SARS-CoV-2 results, as did a friend of hers, whom she had visited in London on February 29, on March 10.

### Case Finding and Epidemiologic Investigations

By March 10, all 16 (100%) of the flight crew and 168 (84%) of the passengers who remained in Vietnam had been traced; 33 (16%) passengers had already transited to other countries. We were able to quarantine, interview, and collect swab specimens for PCR testing from all passengers and crew members who remained in Vietnam. Passengers and crew had traveled on to 15 provinces in Vietnam, ranging from Lao Cai and Cao Bang in the north to Kien Giang in the south.

**Figure 2:**
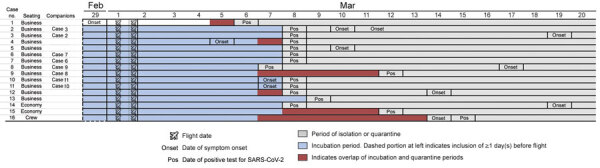
Epidemiologic and clinical timeline for passengers on Vietnam Airlines flight 54, from London, UK, to Hanoi, Vietnam, March 2, 2020, for whom SARS-CoV-2 infection was later confirmed. Because the flight arrived quite early in the morning (5:20 am), we considered the remainder of the day (19 h) to be the day of arrival. Case 14 traveled with a companion who was tested but negative for SARS-CoV-2 infection. SARS-CoV-2, severe acute respiratory syndrome coronavirus 2.

Through these efforts, we identified an additional 15 PCR-confirmed COVID-19 cases, 14 among passengers and 1 among crew members, resulting in a total of 16 confirmed flight-associated cases. Ages of affected persons ranged from 30 to 74 years (median 63.5 years); 9 (>50%) were male, and 12 (75%) were of British nationality (Table 1). Of the 15 persons with flight-associated cases, 12 (80%) had traveled in business class with case 1, and 2 travelers (cases 14 and 15) and 1 flight attendant (case 16) had been in economy class (Figure 1). Among persons in business class, the attack rate was 62% (13/21). Among passengers seated within 2 meters from case 1, which we approximated in business class to be <2 seats away, 11 (92%) were SARS-CoV-2–positive compared with 1 (13%) located >2 seats away (risk ratio 7.3, 95% CI 1.2–46.2) (Table 2). Of the 12 additional cases in business class, symptoms subsequently developed in 8 (67%); median symptom onset was 8.8 days (interquartile range 5.8–13.5) after arrival (Figure 2). None of the additional cases showed COVID-19 symptoms while on board VN54. All 12 additional cases in business class met the definition of probable secondary cases.

**Table 1:** Descriptive epidemiology for 217 passengers and crew on Vietnam Airlines flight 54 from London, UK, to Hanoi, Vietnam, March 2, 2020*

Passenger/crew information	Positive for SARS-CoV-2 by PCR, no. (%)†	Negative for SARS-CoV-2 by PCR, no. (%)
Total	16 (7.4)	201 (92.6)
Age, y		
<18	0	3 (2)
18-49	3 (19)	89 (44)
50-64	4 (25)	80 (40)
>65	9 (56)	29 (14)
Sex		
M	9 (56)	98 (49)
F	7 (44)	103 (51)
Nationality		
British	12 (75)	133 (66)
Vietnamese	3 (19)	31 (15)
Other	1 (6)	37 (18)
Seating location		
Business class	13 (81)	8 (4)
Premium economy class	0	35 (17)
Economy class	2 (13)	143 (71)
Crew members	1 (6)	15 (8)

*Median age, y (interquartile range) was 63.5 (56.0–67.5) for those who were SARS-CoV-2 positive and 51.5 (32.0–60.0) for those who were SARS-CoV-2 negative. SARS-CoV-2, severe acute respiratory syndrome coronavirus 2. †including the probable index case.

**Table 2:** Risk for SARS-CoV-2 infection by seating location among business class passengers on Vietnam Airlines flight 54 from London, UK, to Hanoi, Vietnam, March 2, 2020*

Seating location in relation to index case	Positive for SARS-CoV-2 by PCR, no. (%)†	Negative for SARS-CoV-2 by PCR, no. (%)	Relative risk	Risk ratio (95% CI)
<2 seats away	11 (92)	1 (13)	0.9	7.3 (1.2–46.2)
>2 seats away	1 (8)	7 (88)	0.1	

*SARS-CoV-2, severe acute respiratory syndrome coronavirus 2. †Excluding the index case.

Our investigation did not reveal strong evidence supporting potential SARS-CoV-2 exposure either before or after the flight for any of the additional persons with flight-associated cases other than having traveled on the same flight as case 1 (Appendix). There were 4 traveling companion couples on board, and individuals within each couple sat next to each other in business class. None of the couples or individual cases traveled or stayed with another couple or individual case before the flight or after arrival in Vietnam. Of these case-pairs, 3 (6 persons) were positive for SARS-CoV-2 on the same date: 6 days after arrival in Vietnam.

Among >1,300 close contacts of VN54 passengers and crew members, 5 confirmed cases were identified, 3 of whom were household personnel linked to case 1. The timing of last contact of the remaining 2 confirmed close contacts with their respective flight-associated cases suggests that infection of the flight-associated cases occurred at the same time and that time of infection coincided with the time of the flight (Appendix).

## Discussion

Among the 217 passengers and crew members on a direct flight from London to Hanoi in early March 2020, we identified a cluster of 16 laboratory-confirmed COVID-19 cases. In-depth epidemiologic investigations strongly suggest that 1 symptomatic passenger (case 1) transmitted SARS-CoV-2 infection during the flight to at least 12 other passengers in business class (probable secondary cases).

Case 1 was the only symptomatic person on board and was the only person with a flight-associated case who had established contact with a person with a confirmed case (her sister) during her incubation period. The incubation periods for all persons with confirmed flight-associated cases overlapped with the timing of the flight (Figure 2). Our interviews did not reveal that any of the additional persons with flight-associated cases had been exposed to SARS-CoV-2 before or after the flight during their incubation periods other than having taken the same flight as case 1, nor did they suggest exposure for any of the 4 travel companion couples after the flight (Appendix). Similar intervals between arrival and positive SARS-CoV-2 test results among 3 case-pairs suggest a common exposure event rather than subsequent infection from one partner to the other. Last, we found a clear association between sitting in close proximity to case 1 and risk for infection (Table 2).

In the absence of genomic analysis, we were unable to completely rule out alternative transmission routes. However, all persons with flight-associated cases departed from the United Kingdom (none transited from other countries); and until the departure date of flight VN54, only 23 COVID-19 cases had been recorded in the United Kingdom. Although testing had not been implemented on a large scale nationwide at that time (*19*), community transmission in the United Kingdom was not yet widely established (*20*), making the presence of multiple persons on board incubating the illness unlikely. Similarly, for case 4, who reported having visited India before the United Kingdom during his incubation period, the possibility of preflight transmission remains slim because by March 1, only 3 cases of COVID-19 had been reported in India, although testing in India was still limited (*20*–*22*). Furthermore, none of the 30 colleagues of case 4, who shared the same preflight travel history but were all seated in economy class, were infected (Appendix).

We consider local transmission after arrival in Vietnam unlikely. As of March 1, 2020, only 16 cases of COVID-19 had been reported in Vietnam, and 17 days had passed since the last reported case (case 1 reported here became Vietnam case no. 17) (*18*). At that time, 1,593 persons had tested negative for SARS-CoV-2 infection in Vietnam, and according to official policy at that time, another 10,089 contacts and travelers returning from COVID-19–affected areas overseas were under preemptive quarantine directly at the time of arrival. In early March 2020, there was no evidence of community transmission of SARS-CoV-2 in Vietnam (*18*). We also note that cases 3 and 14 experienced symptom onset 17 days after flight VN54. Whether these cases reflect unusually long incubation periods or symptoms caused by conditions other than COVID-19 is unknown.

The most likely route of transmission during the flight is aerosol or droplet transmission from case 1, particularly for persons seated in business class (*23*). Contact with case 1 might also have occurred outside the airplane at the airport, in particular among business class passengers in the predeparture lounge area or during boarding. Although Vietnam Airlines keeps business class passengers separated from economy class passengers during most procedures before and during the flight, contact with the 2 economy class cases might have occurred after arrival during immigration or at baggage claim. We also note that 2 passengers, in the seats between the 2 cases in economy class, were lost to follow-up. Whether either of these passengers could represent a separate index case in economy class is unknown. 

The role of fomites and on-board surfaces such as tray tables and surfaces in toilets remains unknown. For example, airline crew often use business class toilets while on board, which might explain the case among the crew serving in economy class, for whom no other potential source of infection could be established. Of note, the temporal sequence of symptom onset among cases in economy class and the crew member serving in economy class also allows for the possibility of a second in-flight transmission event, independent of the cluster in business class (Figure 2).

Our study has several limitations. First, we did not have genomic sequencing data available to support our hypothesis of in-flight transmission. However, the conclusiveness and unambiguity of our in-depth epidemiologic upstream and downstream investigations coupled with extensive laboratory testing make us confident of our main findings. Second, we lacked detailed data on activities of the cases while on board (e.g., movements or seat changes, use of toilets, or sharing meals), which might have enabled us to pinpoint the precise route of transmission. Third, our assessment of passengers’ preflight exposure to other confirmed cases relied on interviews only. Fourth, we had no data available on individual passenger use of face masks while on board, which would have enabled a more refined risk analysis. Face masks were neither recommended nor widely used on airplanes in early March, in particular not among travelers from Europe (*24*–*26*), who constituted the majority of passengers on flight VN54. Last, given the delay between arrival and confirmation of the probable index case, no environmental samples could be collected from the airplane.

Our findings have several implications for international air travel, especially because several countries have resumed air travel despite ongoing SARS-CoV-2 transmission. First, thermal imaging and self-declaration of symptoms have clear limitations, as demonstrated by case 1, who boarded the flight with symptoms and did not declare them before or after the flight. Second, long flights not only can lead to importation of COVID-19 cases but also can provide conditions for superspreader events. It has been hypothesized that a combination of environmental factors on airplanes (humidity, temperature, air flow) can prolong the presence of SARS-CoV-2 in flight cabins (*27*). No evidence indicated that the regular air conditioning and exchange system on flight VN54 were malfunctioning. The number of probable secondary cases detected in our study is on the upper end of hypothesized estimations for SARS-CoV-2 transmission on airplanes in the absence of face mask use, although the movement of aerosols and droplets in the specific conditions of a flight cabin remains poorly understood (*27*). A study of a COVID-19 cluster with 16 infected flight passengers from Singapore in February 2020 identified only 1 instance of potential in-flight transmission (*28*). In-flight transmission has been hypothesized but not substantiated sufficiently in a non–peer-reviewed report of a cluster of 10 flight-associated cases in China in February (N. Yang et al., unpub. data, https://www.medrxiv.org/content/10.1101/2020.03.28.20040097v1.full.pdf). In January 2020, no secondary cases were detected after a 15-hour flight to Canada with a symptomatic person with COVID-19 on board (*29*), although contact tracing and monitoring were limited (*30*). Similar results with similar limitations have been reported from flights arriving in France (*31*,*32*) and Thailand (*33*) in January and February. All of these studies limited contact tracing to passengers within 2 rows of the index cases, which could explain why secondary flight-related transmission was not detected by those studies.

The latest guidance from the international air travel industry classifies the in-flight transmission risk as very low (*34*) and recommends only the use of face masks without additional measures to increase physical distance on board, such as blocking the middle seats (*7*,*35*). Our findings challenge these recommendations. Transmission on flight VN54 was clustered in business class, where seats are already more widely spaced than in economy class, and infection spread much further than the existing 2-row (*36*) or 2 meters (*37*) rule recommended for COVID-19 prevention on airplanes and other public transport would have captured. Similar conclusions were reached for SARS-CoV superspreader events on a flight in 2003, in which a high risk for infection was observed for passengers seated farther than 3 rows from the index case (*4*). This finding also concurs with transmission patterns observed for influenza virus (*38*) and is generally in line with the mounting evidence that airborne transmission of SARS-CoV-2 is a major yet underrecognized transmission route (*39*,*40*).

Our findings call for tightened screening and infection prevention measures by public health authorities, regulators, and the airline industry, especially in countries where substantial transmission is ongoing (*37*). Making mask wearing obligatory and making hand hygiene and cough etiquette standard practice while on board and at airports seems an obvious and relatively simple measure (*27*). Blocking middle seats, currently recommended by the airline industry (*7*,*35*), may in theory prevent some in-flight transmission events but seems to be insufficient to prevent superspreading events. Also, systematic testing, quarantine policies, or both, for inbound passengers at arrival might be justified for countries with low levels of community transmission, high risk for case importation, and limited contact tracing capacity (*5*). In Vietnam, for example, as a result of this investigation, national policy was changed toward mandatory testing at arrival irrespective of departure location and 14-day quarantine irrespective of test result or clinical signs/symptoms (*41*). This policy change eliminated the need for resource-intensive contact tracing of flight passengers altogether and enabled detection of another 106 cases among »5,000 passengers on 44 flights until all international flights were halted on March 28. However, given the logistic and economic implications of such policies, developing a quick and reliable point-of-care test that covers the entire infectious period remains paramount.

We conclude that the risk for on-board transmission of SARS-CoV-2 during long flights is real and has the potential to cause COVID-19 clusters of substantial size, even in business class–like settings with spacious seating arrangements well beyond the established distance used to define close contact on airplanes. As long as COVID-19 presents a global pandemic threat in the absence of a good point-of-care test, better on-board infection prevention measures and arrival screening procedures are needed to make flying safe.

Appendix Additional results from epidemiologic investigation of transmission of severe acute respiratory syndrome coronavirus 2 during long flight.
