# Supplementary material for: Transmission of SARS-CoV 2 During Long-Haul Flight
Source: Emerg Infect Dis. 2020 Nov;26(11):2617–24. doi: 10.3201/eid2611.203299 (PMC7588538; doi:10.3201/eid2611.203299)
Supplement: Appendix — Additional results from epidemiologic investigation of transmission of severe acute respiratory syndrome coronavirus 2 during long flight. [file 20-3299-Techapp-s1.pdf]

Article DOI: https://doi.org/10.3201/eid2611.203299

Transmission of Severe Acute Respiratory Syndrome Coronavirus 2 During Long Flight

Appendix

Appendix Table. Summary of epidemiological investigation of additional flight-related cases of flight VN54.

Case no.	Travel history
2 & 3	Case no. 2 (Female, 67) and no. 3 (Male, 74) were life partners from England. After arrival, they stayed in Hanoi for two days. On 4 March, they took a taxi to Quang Ninh – a Northern coast province about 122 miles from Hanoi that is popular for sightseeing. On 5-7 March, case no. 2 and 3 took a sightseeing ship around the island (separate with other cases). On 8 March, they were located, transferred to a local hotel for quarantine and tested positive for SARS-CoV-2 on the same day. None of them was presenting any symptoms of COVID-19 at that time.
4	Case no. 4 (Male, 51) was a Vietnamese government official who had been travelling with a group of 30 other officials on a business trip to India and England since mid-February. He did not experience any COVID-19 symptoms while abroad. On flight VN54 he was sitting in seat 5A, in same row with case no. 1, with no companion. After arrival, he travelled to and from work within Hanoi, and attended several meetings and gatherings. On March 5, he felt fatigue and joint pain in the morning, and became feverish in the evening. On 6 March, after being contacted following the confirmation of case no. 1, he was transferred to a designated hospital for quarantine and monitoring, where he was tested positive for SARS-CoV-2 the next day. None of his work colleagues, who had travelled with him in India and UK but were seated in the economy class of the same flight, tested positive for SARS-CoV-2.
5	Case no. 5 (Male, 58) was a single lone traveler from the UK who stayed for 3 days in Hanoi until 4 March, when he took a day tour to a nearby province with a tour guide (who was later confirmed positive with SARS-CoV-2 with symptoms onset on 7 March). He travelled further to Quang Ninh to take a cruise ship (separate with other cases) from 5-7 March. On 8 March, he was located, transferred to a local hotel for quarantine and tested positive for SARS-CoV-2 on the same day. He was asymptomatic at that time.
6 & 7	Case no. 6 (Male, 66) and case no.7 (Male, 60) were friends from UK and travelled together to Da Nang, a large coastal city in Southern Middle area of Vietnam, by flight on 4 March, after a one-day stay in Hanoi, where they had close contact with a saleswoman (who were later confirmed positive with COVID-19 with symptoms onset on 6 March) on the same day during a 15-minute conversation. Since then, the cases travelled together nearby Da Nang. On 7 March, after all hotels in Vietnam were alerted about unfolding outbreak, they were instructed to self-isolate in their hotel rooms. At the time of testing on 8 March, none of them were symptomatic.
8 & 9	Case no. 8 (Female, 66) and case no. 9 (Male, 71) were life partners from the UK. After arrival, they stayed in Hanoi until 6 March, when they travelled together by flight to Hue, a city in central Vietnam, where they were directly asked to self-quarantine in their room by alerted hotel staffs. On 7 March, samples were collected and later that day returned positive for case no. 8, while no. 9 tested negative initially. Both cases remained quarantined/isolated, and on 12 March, case no. 9 was confirmed positive as well.
10 & 11	Case no. 10 (Female, 70) and case no. 11 (Male, 69) were life partners from the UK, they stayed in Hanoi for two days after arrival. On 4 March, they took a night train to Lao Cai, a Northwest mountainous province of Vietnam bordering China. After checking into a local hotel on 5 March, they took a tour bus to Sa Pa, a nearby small mountainous town where they stayed in a hotel until 7 March, when local health staffs managed to locate and isolate them. They started experiencing cough and fever, and tested positive on the same day.
12	Case no. 12 (Female, 66) was a single traveler from the UK. She had already transited to Cambodia on 4 March after a two-day stay in Hanoi. She was identified by the Cambodian health authorities and tested for SARS-CoV-2 on 7 March. Her testing status and findings from epidemiological investigation was obtained from the Cambodia Center of Disease Control.
13	Case no. 13 (Male, 49) was a single traveler from the UK travelling to Da Nang on 6 March after his four-day stay in Hanoi. A day later, he was instructed to self-isolate in his hotel rooms after all hotels in Vietnam were alerted with the folding outbreak. At the time of testing, he was asymptomatic.
14	Case no. 14 (Female, 50) and her partner (who remained negative throughout) were both UK nationals and travelled in economy class. They travelled to Quang Ninh after arrival to Hanoi on 2 March. They took a cruise ship tour on 4-5 March (separate with other cases), and subsequently stayed in their hotel from 5-8 March. On 8 March, they were located, transferred to a local hotel for quarantine and case no. 14 tested positive for SARS-CoV-2 on the same day. At the time of testing, she was asymptomatic.
15	Case no. 15 (Male, 58) traveler from the UK in economy class. He travelled by flight to Da Nang on 5 March from Hanoi. In Da Nang, he travelled to surrounding cities until being contacted by his hotel staff on 7 March and was instructed to self-isolate in his hotel. At the time of testing, he was asymptomatic.
16	Case no. 16 (Female, 30) was a flight crew member serving in economy class. She remained in a common dormitory for Vietnam Airline crew member near Noi Bai Airport, Hanoi since arrival to 6 March. Before VN54 flight, she had served as an attendant on Vietnam Airline international flights on a daily basis to France, UK, Japan and South Korea since January 2020. She was asymptomatic when tested and put into quarantine on 7 March. On 13 March, when experiencing cough and fever, she was confirmed positive for SARS-CoV-2 the next day.

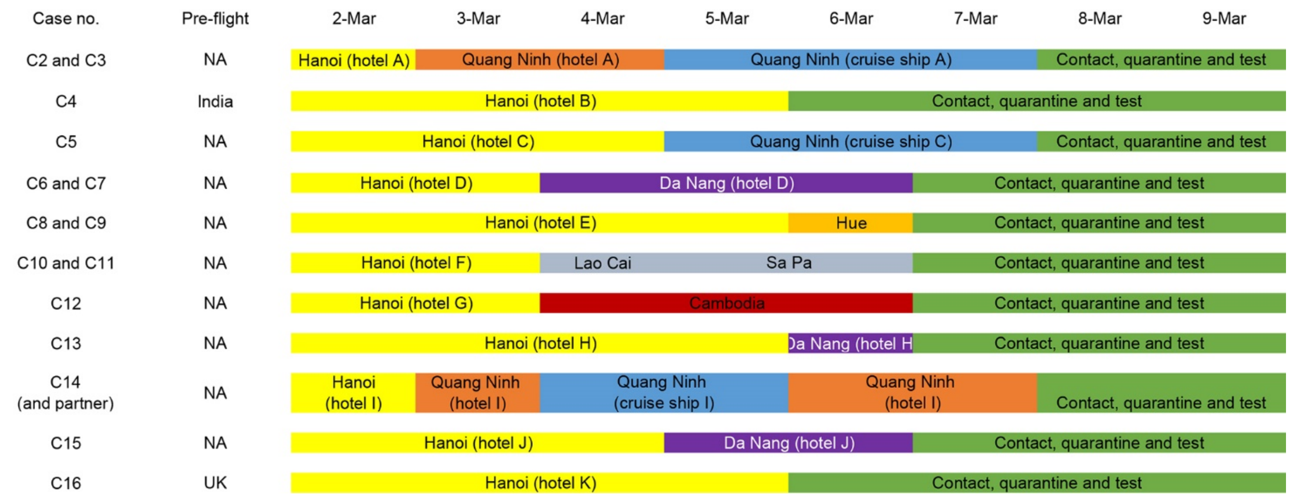

Appendix Figure. Summary of epidemiological investigation of additional flight-related cases of flight VN54.
